# Precrec: fast and accurate precision–recall and ROC curve calculations in R

**DOI:** 10.1093/bioinformatics/btw570

**Published:** 2016-09-01

**Authors:** Takaya Saito, Marc Rehmsmeier

**Affiliations:** 1 Computational Biology Unit, Department of Informatics, University of Bergen, Bergen, Norway; 2 Integrated Research Institute (IRI) for the Life Sciences and Department of Biology, Humboldt-Universität zu Berlin, Berlin, Germany

## Abstract

**Summary:**

The precision–recall plot is more informative than the ROC plot when evaluating classifiers on imbalanced datasets, but fast and accurate curve calculation tools for precision–recall plots are currently not available. We have developed Precrec, an R library that aims to overcome this limitation of the plot. Our tool provides fast and accurate precision–recall calculations together with multiple functionalities that work efficiently under different conditions.

**Availability and Implementation:**

Precrec is licensed under GPL-3 and freely available from CRAN (https://cran.r-project.org/package=precrec). It is implemented in R with C ++.

**Supplementary information:**

Supplementary data are available at *Bioinformatics* online.

## 1 Introduction

The recent rapid advances of molecular technologies have increased the importance of developing efficient and robust algorithms to handle large amounts of data in various fields of bioinformatics. Binary classifiers are mathematical and computational models that have successfully solved a wide range of life science problems with huge volumes of data produced from high-throughput experiments (Saito and Rehmsmeier, 2015). The Receiver Operating Characteristics (ROC) plot is the most popular performance measure for the evaluation of binary classification models. Its popularity comes from several well-studied characteristics, such as intuitive visual interpretation of the curve, easy comparisons of multiple models, and the Area Under the Curve (AUC) as a single-value quantity (Fawcett, 2006). Nonetheless, the intuitive visual interpretation can be misleading and potentially result in inaccurate conclusions caused by a wrong interpretation of specificity when the datasets are imbalanced. Imbalanced data naturally occur in life sciences. For instance, the majority of the datasets from genome-wide studies, such as microRNA gene discovery, are heavily imbalanced (Saito and Rehmsmeier, 2015). The precision–recall plot is an ROC alternative and can be used to avoid this potential pitfall of the ROC plot (He and Garcia, 2009; Saito and Rehmsmeier, 2015).

Although some performance evaluation tools offer the calculation of precision–recall curves, they tend to underestimate several important aspects. One of these aspects is that any point on an ROC curve has a one-to-one relationship with a corresponding point on a precision–recall curve. To satisfy this relationship, precision–recall curves require non-linear interpolations to connect two adjacent points, unlike the simple linear interpolations of ROC curves (Davis and Goadrich, 2006). This non-linear interpolation is further developed in closely connected areas, such as calculations of AUC scores and confidence interval bands (Boyd *et al.*, 2013; Keilwagen *et al.*, 2014). Nonetheless, only a limited number of tools can produce non-linear interpolations of precision–recall curves (Davis and Goadrich, 2006; Grau *et al.*, 2015), and they usually come with high computational demands. Moreover, tools that are specific to precision–recall calculations tend to lack support for pre- and post-processing such as handling tied scores and calculating confidence interval bands, whereas some ROC-specific tools provide multiple functionalities (Robin *et al.*, 2011). We have developed Precrec, a tool that offers fast and accurate precision–recall calculations with several additional functionalities. Our comparison tests show that Precrec is the only tool that performs fast and accurate precision–recall calculations under various conditions.

## 2 Implementation

We separated Precrec into several modules according to their functions, and optimized each module with respect to processing time and accuracy. Specifically, we focused on the following six aspects to achieve high accuracy and multiple functionalities:

1. Calculation of correct non-linear interpolations.
2. Estimation of the first point, which is necessary when the precision value becomes undefined due to no positive predictions.
3. Use of score-wise threshold values instead of fixed bins.
4. Integration of other evaluation measures, such as ROC and basic measures from the confusion matrix.
5. Handling of multiple models and multiple test sets.
6. Addition of pre- and post-process functions for simple data preparation and curve analysis.

The aspects 1–3 are related to correct curve calculations. The remaining aspects pertain to the other evaluation measures and features that Precrec offers. Precrec concurrently calculates ROC and precision–recall curves together with their AUCs. It can also calculate several basic evaluation measures, such as error rate, accuracy, specificity, sensitivity and positive predictive value. Moreover, Precrec can directly accept multiple models and multiple test sets. For instance, it automatically calculates the average curve and the confidence interval bands when multiple test sets are specified. Precrec also has powerful features for data preparation. For instance, it offers several options for handling tied scores and missing values.

To speed up calculations in the Precrec modules, we first tried to optimize only in R. We replaced some R code with C ++ code when it was difficult to solve low-performance issues in R.

## 3 Results

**Fig. 1:**
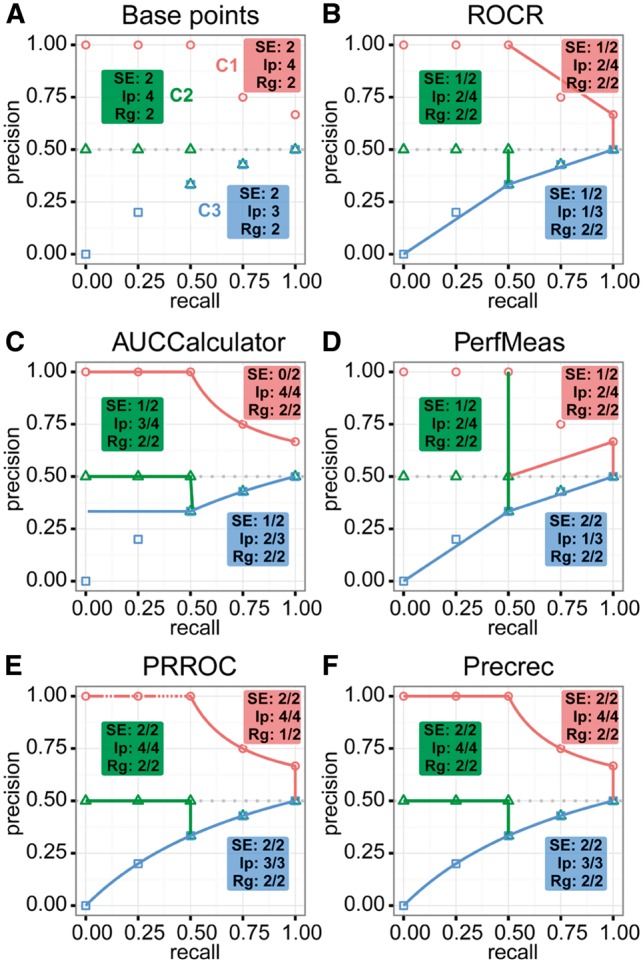
Results of evaluating precision–recall curves calculated by five different tools for three test sets – C1, C2 and C3. (**A**) The plot shows manually calculated points for C1 (red), C2 (green) and C3 (blue). Each test set contains three different test categories: SE (start and end positions), Ip (intermediate position and interpolation) and Rg (x and y ranges). In addition, each category has 3–5 individual test items. The remaining plots show the calculated curves with successes/total per category for (**B**) ROCR, (**C**) AUCCalculator, (**D**) PerfMeas, (**E**) PRROC and (**F**) Precrec

For the evaluation of Precrec, we have developed prcbench, an R library that serves as a compact testing workbench for the evaluation of precision–recall curves (available on CRAN). We have also compared our tool with four other tools that can calculate precision–recall curves: ROCR (Sing *et al.*, 2005), AUCCalculator (Davis and Goadrich, 2006), PerfMeas (available on CRAN) and PRROC (Grau *et al.*, 2015). The workbench provides two types of test results: the accuracy of the curves (Fig. 1) and the benchmarking of processing time (Table 1).

**Table 1:** Benchmarking results of the five tools in millisecond

Tool	Curve	AUC	NL	100	1000	1 million
ROCR	Yes	No	No	5.4	6.8	(2.6 s)
AUCCalculator	Yes	Yes	Yes	105	216	(33 min)
PerfMeas	Yes	Yes	No	0.2	0.4	763
PRROC	Yes	Yes	Yes	348	(74 sec)	(123 days)^a^
PRROC (step=1)	Yes	Yes	No	7.9	96	(6.3 hrs)^a^
PRROC (AUC)	No	Yes	Yes	23.7	236	(4 min)
Precrec	Yes	Yes	Yes	6.4	6.8	463

**Tool**: We performed PRROC (step = 1) with minStepSize = 1 and PRROC (AUC) without curve calculation. **Curve:** curve calculation. **AUC:** AUC calculation. **NL:** non-linear interpolation. **100**, **1000**, **1 million:** test dataset size. We tested each case 10 times and calculated the average (mean) processing time. The measurement unit is millisecond unless indicated otherwise.

a We tested only once for these cases.

### 3.1 Precrec calculates accurate precision–recall curves

Figure 1A shows the base points of three tests sets – C1, C2 and C3. The tests are based on these pre-calculated points through which correctly calculated curves must pass. Each test set contains three categories. SE is for checking the correct curve elongation to the start and the end points. Ip is for correct curve calculations both on the intermediate points and interpolations. Rg is for *x* and *y* ranges; it is less important than the other two categories, but incorrect ranges may cause graph plotting issues. The results show that ROCR, AUCCalculator and PerfMeas (Fig. 1B–D) have inconsistent starting points. Of these three, only AUCCalculator applies non-linear interpolations. Both PRROC and Precrec (Fig. 1E, F) calculate correct curves on C2 and C3, but only Precrec calculates a correct curve for C1, whereas PRROC fails on this set by providing several precision values that are larger than 1 by around 1E-15 in our test environment (indicated by a dotted curve in Figure 1E; see Supplementary methods and results for details).

### 3.2 Precrec uses additional support points for non-linear interpolation and confidence intervals

Precrec relies on additional support points for non-linear interpolation between two adjacent points and offers an option (x_bins) that associates with the number of support points for the whole curve, with the default value being 1000. For instance, the distances between two support points are consistent and respectively 0.5 and 0.001 when x_bins are 2 and 1000. Precrec performs linear interpolation when x_bins is 1. Moreover, this approach enables us to calculate the average curve with confidence interval bands when multiple test datasets are specified.

### 3.3 Precrec provides fast calculations regardless of dataset sizes

Table 1 shows the benchmarking result of processing time for the five tools. All tools perform reasonably well on small (100 items) and medium (1000 items) datasets, but only Precrec appears to be practically useful for calculating accurate non-linear interpolations (NL:Yes) on large (1 million items) datasets (see Supplementary methods and results for details).

### 3.4 Precrec calculates AUCs with high accuracy

Precrec uses the trapezoidal rule to calculate AUC scores. If a different number of support points is specified, the score changes accordingly. We also analyzed the accuracy of AUC scores by using randomly generated datasets. AUC scores appear to be very similar across the tools especially for large datasets. PerfMeas calculates AUC scores that are slightly different from the others, but the differences are small (see Supplementary methods and results for details). The results also show that there are only small differences between linear and non-linear AUCs. Nonetheless, correct non-linear interpolation can be useful when a dataset contains distantly separated adjacent points.

### 3.5 Datasets with class imbalance and tied scores may require non-linear interpolation

Non-linear interpolation is important when two adjacent points are distantly separated. Such a separation usually occurs when the dataset size is small. Nonetheless, it may even occur for large datasets, for instance, if a dataset is heavily imbalanced or contains a number of tied scores (see Supplementary methods and results for details). Hence, it is useful to provide non-linear calculations regardless of the dataset size.

### 3.6 Precrec concurrently calculates ROC curve

ROC and precision–recall curves have a number of aspects in common, and it is sometimes demanded to analyze both curves. Precrec calculates both curves and their AUCs by default.

## 4 Summary

The precision–recall plot is more informative than the ROC plot when evaluating classifiers on imbalanced datasets. Nevertheless, most performance evaluation tools focus mainly on the ROC plot. We have developed a performance evaluation library that works efficiently with various types of datasets and evaluation measures. In summary, Precrec is a powerful tool which provides fast and accurate precision–recall and ROC calculations with various functionalities.

*Conflict of Interest*: none declared.

## Supplementary Material

Supplementary Data
