# Supplementary material for: Precrec: fast and accurate precision–recall and ROC curve calculations in R
Source: Bioinformatics. 2016 Sep 1;33(1):145–7. doi: 10.1093/bioinformatics/btw570 (PMC5408773; doi:10.1093/bioinformatics/btw570)
Supplement: Supplementary Data [file btw570_supp.pdf]

Supplementary data

Precrec: fast and accurate precision-recall and ROC curve calculations in R

Takaya Saito and Marc Rehmsmeier

Contents

Supplementary methods

1. Tools for precision-recall calculations ··· 2
2. Validation of precision-recall curves ··· 2
3. Benchmarking of processing time ··· 3
4. Preparation for AUC analysis ··· 4
5. Data preparation of imbalanced datasets ··· 4
6. Data preparation of tied scores ··· 5
7. Test environment ··· 5

Supplementary results

1. Precision-recall curve validations on C1, C2, and C3 ··· 6
2. Benchmarking of processing time on randomly generated test sets ··· 7
3. AUC analysis ··· 8
4. Imbalanced data analysis with large datasets ··· 9
5. Analysis on tied scores of a large dataset ··· 9

References ··· 10

Supplementary methods

1. Tools for precision-recall calculations

We compared Precrec (Saito and Rehmsmeier, 2016b) with four other tools that can calculate precision-recall curves: ROCR (Sing *et al.*, 2005), AUCCalculator (Davis and Goadrich, 2006), PerfMeas (Valentini and Re, 2016), and PRROC (Grau *et al.*, 2015) (Table S1).

Table S1. Four tools for the comparisons with Precrec.

Tool	Version	Language	URL
ROCR	1.0-7	R	https://cran.r-project.org/package=ROCR
AUCCalculator	0.2	Java	http://mark.goadrich.com/programs/AUC
PerfMeas	1.2.1	R	https://cran.r-project.org/package=PerfMeas
PRROC	1.1	R	https://cran.r-project.org/package=PRROC

2. Validation of precision-recall curves

To validate whether the tools can compute correct precision-recall curves, we manually created three test datasets C1, C2, and C3 (Table S2).

Table S2. Test datasets C1, C2, and C3.

Dataset	Row ID	Score	Label
C1	1	3	1
	2	2	0
	3	2	1
	4	1	0
C2	1	3	1
	2	3	0
	3	1	1
	4	2	0
C3	1	2	1
	2	4	0
	3	3	0
	4	1	1

We then manually calculated precision-recall curves with interpolated precision values following the method proposed by Davis and Goadrich (Davis and Goadrich, 2006) (Tables S3-S5).

Table S3. Recall and precision values for C1.

Ranks[1]	TP[2]	FP[3]	Recall	Precision	Interpolated
1	0	0	0	1[†]	No
	0.5		0.25	1	Yes
2	1	0	0.5	1	No
3	1.5	0.5	0.75	0.75	No
4	2	1	1	0.67	No
5	2	2	1	0.5	No

[1]Ranks are calculated from scores. [2,3]TP and FP are the number of true positives and false positives from the confusion matrix, respectively. [†]Value is estimated from the adjacent precision value since the original value is undefined.

Table S4. Recall and precision values for C2.

Ranks[1]	TP[2]	FP[3]	Recall	Precision	Interpolated
1	0	0	0	0.5[†]	No
2	0.5	0.5	0.25	0.5	No
3	1	1	0.5	0.5	No
4	1	2	0.5	0.33	No
	1.5		0.75	0.43	Yes
5	2	2	1	0.5	No

[1]Ranks are calculated from scores. [2,3]TP and FP are the number of true positives and false positives from the confusion matrix, respectively. [†]Value is estimated from the adjacent precision value since the original value is undefined.

Table S5. Recall and precision values for C3.

Ranks[1]	TP[2]	FP[3]	Recall	Precision	Interpolated
1	0	0	0	0[†]	No
2	0	1	0	0	No
3	0	2	0	0	No
	0.5		0.25	0.2	Yes
4	1	2	0.5	0.33	No
	1.5		0.75	0.43	Yes
5	2	2	1	0.5	No

[1]Ranks are calculated from scores. [2,3]TP and FP are the number of true positives and false positives from the confusion matrix, respectively. [†]Value is estimated from the adjacent precision value since the original value is undefined.

We tested the five tools on C1, C2, and C3 and evaluated whether they could produce correct recall and precision values. We used the prcbench tool (Saito and Rehmsmeier, 2016a) for testing five important aspects of precision-recall curves on C1, C2, and C3 (Table S6).

Table S6. Test categories and items for evaluating precision-recall curves.

Category	Test item	Description	# tests
SE	fpoint	Check the first point. It must be correctly estimated when undefined.	1
	epoint	Check the end point. It must be the point calculated as T / (T + N).	1
Ip	int_pts	Check the intermediate points.	C1: 4<br>C2: 4<br>C3: 3
Rg	x_range	Evaluate the range of recall values. The range must be [0, 1].	1
	y_range	Evaluate the range of precision values. The range must be [0, 1].	1

3. Benchmarking of processing time

We tested the processing time of the five tools with randomly generated test datasets. We used the prcbench tool to create random scores with five different dataset sizes (Table S7).

Table S7. Datasets for benchmarking

Dataset name	Data size	# of positives	# of negatives
100	100	50	50
1k	1000	500	500
10k	10000	5000	5000
100k	100000	50000	50000
1m	1000000	500000	500000

Some tools calculate both ROC and precision-recall curves together with the corresponding AUC scores (Table S8). Moreover, the PRROC tool has an effective approach to calculate the area under the precision-recall curve. We were also interested in checking the process time with different parameters of PRROC. Hence, we tested PRROC with three different parameter configurations.

Table S8. Tool configurations for benchmarking

Tool	Curve[1]	AUC[2]	Parameters
ROCR	PRC	-	measure=”prec”, x.measure="rec"
AUCCalculator	PRC & ROC	PRC & ROC	fileType: list
PerfMeas	PRC	PRC	comp.precision = TRUE
PRROC	PRC	PRC	curve = TRUE, minStepSize = 0.01
PRROC (step=1)	PRC	PRC	curve = TRUE, minStepSize = 1
PRROC (AUC)	-	PRC	curve = FALSE
Precrec	PRC & ROC	PRC & ROC	(default)

[1,2]PRC indicates precision-recall curves.[1]The type of curves the tool calculates. [2]The type of AUCs the tool calculates.

We used the prcbench tool to iterate all combinations of tools and test sets 10 times and calculated the average (mean) processing time for each tool and test set.

4. Preparation for AUC analysis

We tested four tools except for ROCR since it provide no AUC scores for precision-recall curves. We generated three different sizes of datasets, 50, 100, and 1000. We repeat the AUC calculation process 100 times and subsequently calculated the mean and the standard errors of the generated AUC scores. Datasets were randomly generated each iteration, and they were all balanced datasets.

5. Data preparation of imbalanced datasets

We used three different datasets to analyze the difference between linear and non-linear interpolation.

1. Balanced: positives 500 & negatives 500

2. Imbalanced 1: positives 100 & negatives 900
3. Imbalanced 1: positives 10 & negatives 990

We randomly generated the scores by sampling from the distributions of positives and negatives as N(3, 1) and N(1, 1). We used Precrec to produce precision-recall plots.

6. Data preparation of tied scores

We created a dataset with tied scores by randomly sampling 1000 positives and 1000 negatives as N(0.25, 0.5) and N(0, 0.5). We replaced all values smaller than 0 with 0 and all values larger than 1 with 1. We used ROC and Precrec to compare between linear and non-linear curves.

7. Test environment

We used our local machine for all tests and evaluations, a Linux workstation (CentOS 6.7) with Intel i7 4 cores (8 threads; 3.40GHz) and 16 GB RAM. We ran all tests with using a single core.

Supplementary results

1. Precision-recall curve validations on C1, C2, and C3

Table S9 shows the summarized scores of precision-recall curve validations on C1, C2, and C3.

Table S9. Summarized validation scores of C1, C2, and C3

Tool	Score (C1)	Score (C2)	Score (C3)	Total score
ROCR	5/8	5/8	4/7	14/23
AUCCalculator	6/8	6/8	5/7	17/23
PerfMeas	5/8	5/8	5/7	15/23
PRROC	7/8	8/8	7/7	22/23
Precrec	8/8	8/8	7/7	23/23

In addition, Table S10 shows the detailed test results of precision-recall curve validations.

Table S10. Detailed test results of C1, C2, and C3

Tool	Test item	# of successes		
		C1	C2	C3
ROCR	x_range	1	1	1
	y_range	1	1	1
	fpoint	0	0	0
	int_pts	2	2	1
	epoint	1	1	1
AUCCalculator	x_range	1	1	1
	y_range	1	1	1
	fpoint	0	0	0
	int_pts	4	3	2
	epoint	0	1	1
PerfMeas	x_range	1	1	1
	y_range	1	1	1
	fpoint	0	0	1
	int_pts	2	2	1
	epoint	1	1	1
PRROC	x_range	1	1	1
	y_range	0	1	1
	fpoint	1	1	1
	int_pts	4	4	3
	epoint	1	1	1
Precrec	x_range	1	1	1
	y_range	1	1	1
	fpoint	1	1	1
	int_pts	4	4	3
	epoint	1	1	1

2. Benchmarking of processing time on randomly generated test sets

Tables S11-15 show the detailed benchmark results of the 100, 1k, 10k, 100k, and 1m test sets.

Table S11. Benchmark result of the 100 dataset (in milliseconds)

Tool	min	lq	mean	median	uq	max
ROCR	5.19	5.24	5.35	5.29	5.39	5.87
AUCCalculator	99.18	102.33	104.9	104.88	105.76	118.21
PerfMeas	0.12	0.13	0.2	0.14	0.2	0.61
PRROC	302.26	304.43	348.34	322.74	396.91	419.03
PRROC (step=1)	6.61	6.97	7.88	7.90	8.82	9.08
PRROC (AUC)	22.42	22.86	23.74	23.4	24.74	25.24
Precrec	6.21	6.25	6.37	6.33	6.4	6.75

All values are rounded to two decimal places.

Table S12. Benchmark result of the 1k dataset (in milliseconds)

Tool	min	lq	mean	median	uq	max
ROCR	6.47	6.56	6.75	6.75	6.82	7.15
AUCCalculator	206	212	217	219	221	227
PerfMeas	0.25	0.28	0.38	0.31	0.38	0.88
PRROC	65359	75026	74485	75212	75965	76639
PRROC (step=1)	79.81	86.63	96.01	88.21	90.92	175
PRROC (AUC)	226	226	236	227	228	313
Precrec	6.07	6.24	6.75	6.47	7.11	8.66

Values greater than 100 are rounded to integer values. The remaining values are rounded to two decimal places.

Table S13. Benchmark result of the 10k dataset (in milliseconds)

Tool	min	lq	mean	median	uq	max
ROCR	17.69	18.17	19.44	19.58	19.65	21.71
AUCCalculator	623	644	675	674	710	739
PerfMeas	1.79	1.83	1.89	1.87	1.93	2.07
PRROC	3173144	3523302	4965855	5451419	5798057	7701460
PRROC (step=1)	2683	2710	2811	2785	2896	3020
PRROC (AUC)	2300	2302	2322	2305	2360	2370
Precrec	8.88	8.96	9.54	9.00	10.04	11.35

Values greater than 100 are rounded to integer values. The remaining values are rounded to two decimal places.

Table S14. Benchmark result of the 100k dataset (in milliseconds)

Tool	min	lq	mean	median	uq	max
ROCR	165	168	176	170	172	239
AUCCalculator	102401	10374	10410	10415	10478	10620
PerfMeas	24.61	25.35	26.17	25.95	26.10	29.31
PRROC	204392863	209714530	212283055	211126882	212550009	229970968
PRROC (step=1)	554701	709282	697908	712767	716595	719552
PRROC (AUC)	23246	23340	23420	23400	23517	23681
Precrec	35.36	36.59	38.7	39.64	40.55	41

Values greater than 100 are rounded to integer values. The remaining values are rounded to two decimal places.

Table S15. Benchmark result of the 1m dataset (in milliseconds)

Tool	min	lq	mean	median	uq	max
ROCR	2510	2556	2603	2590	2625	2778
AUCCalculator	1905023	1912508	1961509	1929234	1949094	2116694
PerfMeas	689	702	763	710	727	1154
PRROC†	-	-	-	-	-	-
PRROC (step=1)†	-	-	-	-	-	-
PRROC (AUC)	243524	244681	247480	247656	249759	252026
Precrec	399	434	463	480	487	490

Values are rounded to integer values. †Tool was not tested by prcbench on this dataset.

3. AUC analysis

Table S16 shows the mean and standard error (SE) of the AUC scores calculated by the four tools. Both PRROC and Precrec used linear and non-linear (NL) methods to calculate the AUCs.

The AUC scores appear to be similar among all tools regardless of linear or non-linear methods. PerfMeas is slightly different from the others, but the differences are small.

Table S16. AUC scores

Tool	50		100		1000	
	mean	SE	mean	SE	mean	SE
AUCCalculator	0.8425627	0.005343464	0.8410968	0.003812543	0.8322225	0.001325158
PerfMeas	**0.8025627**	0.005343464	**0.8210968**	0.003812543	**0.8302225**	0.001325158
PRROC	0.8426215	0.005338885	0.8410968	0.003812543	0.8322225	0.001325158
Precrec	0.8426215	0.005338887	0.8410968	0.003812543	0.8322225	0.001325158
PRROC (NL)	0.8425627	0.005343464	0.8411109	0.003811922	0.8322227	0.001325155
Precrec (NL)	0.8425627	0.005343464	0.8411109	0.003811924	0.8322226	0.001325156

4. Imbalanced data analysis with large datasets

Figure S1 shows precision-recall curves calculated from three different datasets. We used Precrec to calculate both linear and non-linear interpolations. The precision-recall curves of linear and non-linear are almost identical for the balanced dataset and the first imbalanced dataset with 100 positives and 900 negatives. Nonetheless, the difference is noticeable for the second imbalanced dataset with 10 positives and 990 negatives especially for small recall values between 0 and 0.25.

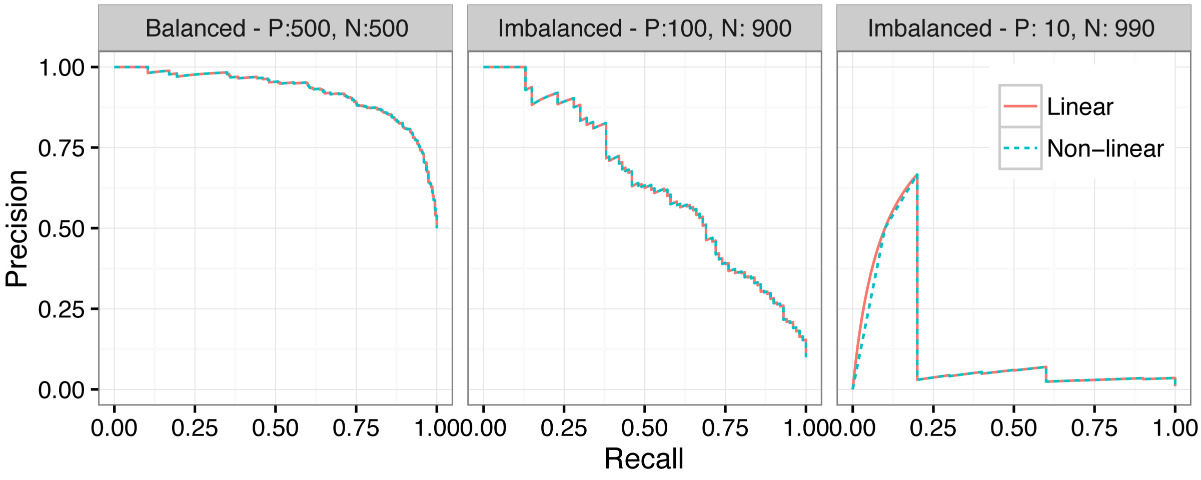

Fig. S1. Precision-recall curves on balanced and imbalanced datasets.

5. Analysis on tied scores of a large dataset

Fig. S2 shows the result of Precrec and ROCR on a test dataset with 1000 positives and 1000 negatives. Fig. S2A indicates the range of scores is [0, 1], and the data set contains a number of 0s and 1s. Fig. S2B shows there are differences between linear (ROCR) and non-linear (Precrec) interpolation.

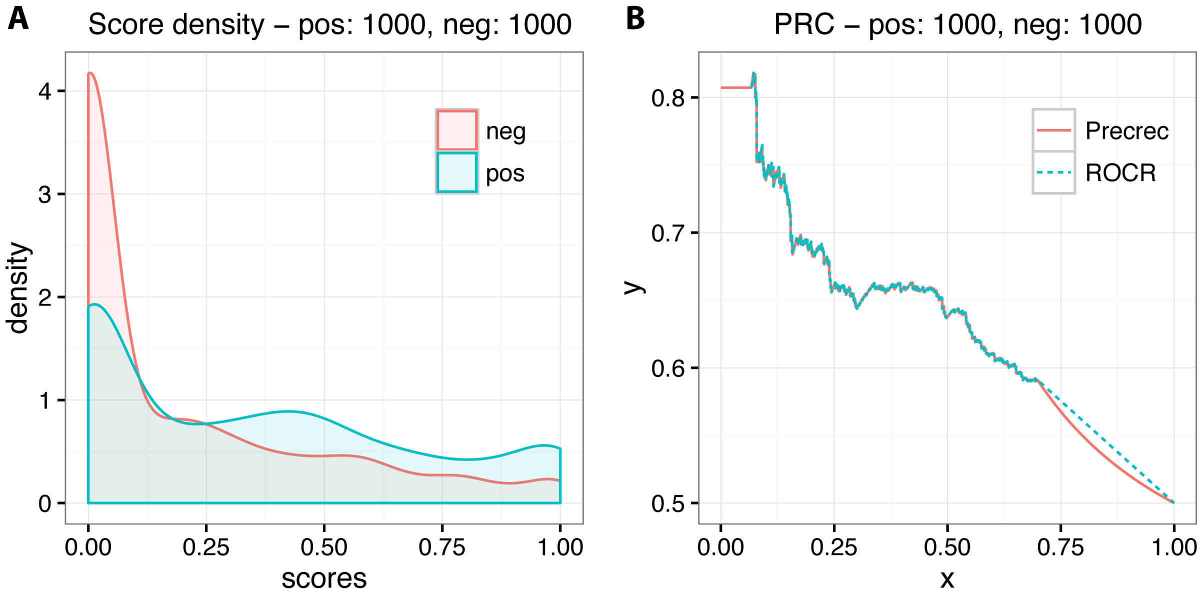

Fig. S2. Analysis of Precrec and ROCR on a dataset with tied scores.

References

Davis, J. and Goadrich, M. (2006) The relationship between Precision-Recall and ROC curves. Proceedings of the 23rd international conference on Machine learning, 233-240.

Grau, J. *et al.* (2015) PRROC: computing and visualizing precision-recall and receiver operating characteristic curves in R. *Bioinformatics*, **31**, 2595-2597.

Saito, T. and Rehmsmeier, M. (2016a) prcbench: Testing Workbench for Precision-Recall Curves. *https://cran.r-project.org/package=prcbench.*

Saito, T. and Rehmsmeier, M. (2016b) precrec: Calculate Accurate Precision-Recall and Receiver Operator Characteristics Curves. *https://cran.r-project.org/package=precrec.*

Sing, T. *et al.* (2005) ROCR: visualizing classifier performance in R. *Bioinformatics*, **21**, 3940-3941.

Valentini, G. and Re, M. (2016) PerfMeas: Performance Measures for ranking and classification tasks. *https://cran.r-project.org/package=PerfMeas.*
